# Supplementary material for: Collateral Vessels Have Unique Endothelial and Smooth Muscle Cell Phenotypes
Source: Int J Mol Sci. 2019 Jul 24;20(15):3608. doi: 10.3390/ijms20153608 (PMC6695737; doi:10.3390/ijms20153608)
Supplement: Supplementary file 1 [file ijms-20-03608-s001.pdf]

Supplemental file

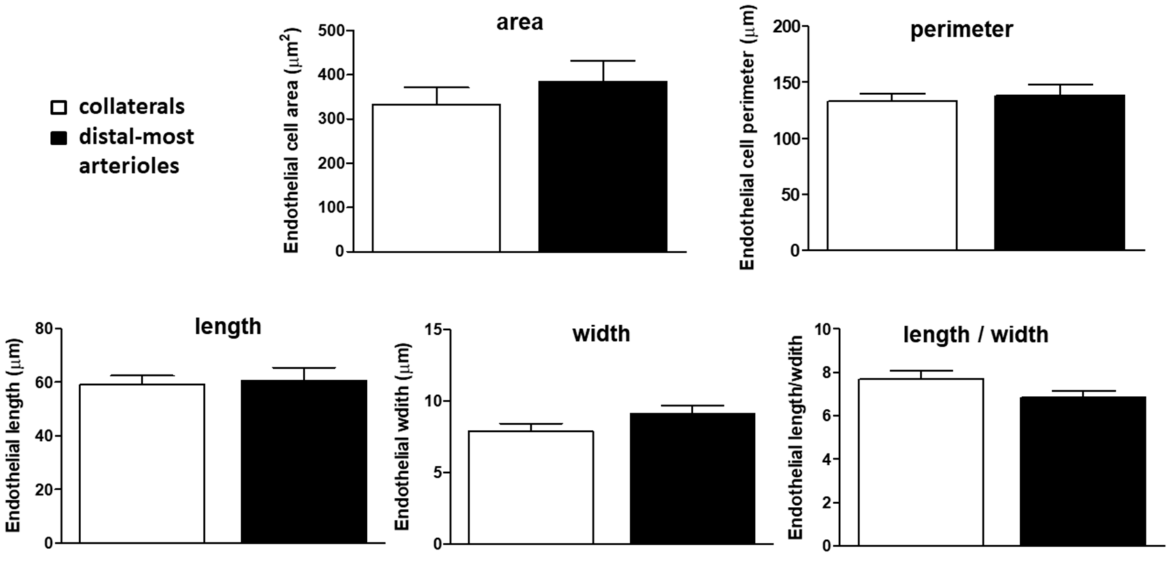

Figure S1. Dimensions of collateral endothelial cells are not significantly different from those of nearby distal-most arterioles. Data obtained from SEM samples shown in Figures 1, 2D (2–3 of each vessel type per mouse). p =0.08 for length/width, 2-sided t-tests.

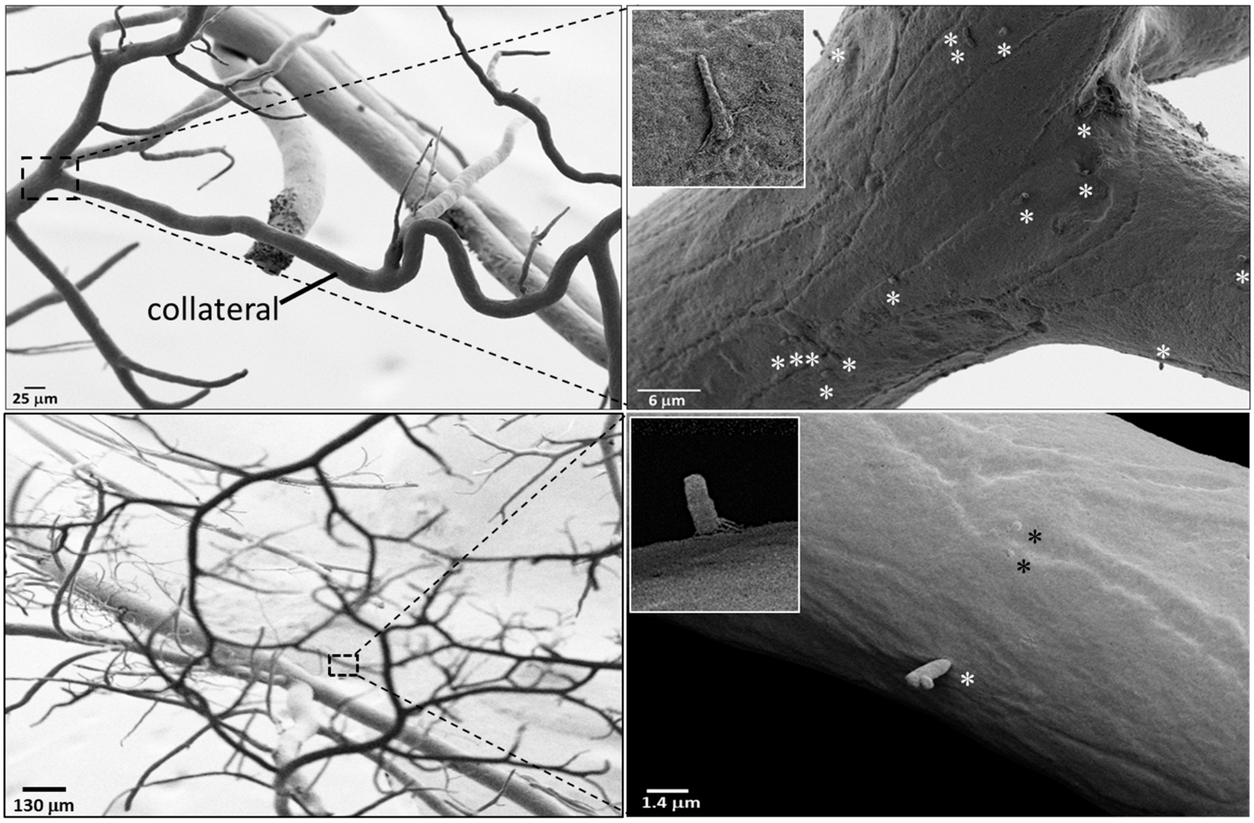

Figure S2. Like collaterals, distal arterioles that either continue-on as collaterals (top panels) or do not (bottom panels) also express primary cilia (stars; see also Figures 1, 4). Scanning electron micrographs of corrosion casts of arterial vessels, fixed at maximal dilation, overlying the watershed zone between the ACA and MCA trees obtained from two mice (top vs bottom panels) different from mice shown in Figures 1, 3. Insets in right panels show single ~2 μm primary cilia invaginations.
